# Next-generation sequencing yields the complete mitogenome of red-crowned crane (*G. japonensis*)

**DOI:** 10.1080/23802359.2018.1467220

**Published:** 2018-04-28

**Authors:** Rong Hua, Duoying Cui, Jia Liu, Yuyan You, Ting Jia

**Affiliations:** Beijing Key Laboratory of Captive Wildlife Technologies, Beijing Zoo, Beijing, China

**Keywords:** Complete mitogenome, red-crowned crane, next-generation sequencing

## Abstract

In this study, the complete mitogenome sequence of red-crowned crane (*G. japonensis*) has been decoded by next-generation sequencing and genome assembly. The assembled mitogenome, consisting of 16,727 bp, has unique 14 protein-coding genes (PCGs), 22 transfer RNAs, and two ribosomal RNAs genes. The complete mitogenome provides essential and important DNA molecular data for further phylogenetic and evolutionary analysis for red-crowned crane phylogeny.

The red-crowned crane (*G. japonensis*) is one of the rarest crane species and has been classified as ‘endangered’ on the International Union for Conservation of Nature (IUCN) Red List (IUCN 2014). The current population is estimated to be 2750 individuals (Bird Life International 2015). A large proportion of the migratory population migrates from northeastern China to Yancheng in late October and overwinters in the Yancheng Biosphere Reserve, China, itself the largest wintering area for the migratory population, until early March (Cui et al. 2017). Red-crowned cranes are omnivorous, whose diet includes rice, grain, wheat seedlings, shellfish, fish, shrimp, crabs, snails, seepweed seed and leaf, and reed roots (Liu et al. 2016). As Yancheng is located in a climatic zone with temperature and humidity suitable for mold growth and mycotoxin production, it is likely that some of these foods are contaminated by mycotoxins. Such a situation might have a negative impact on the health and reproduction of these cranes, and thus potentially threatens the population. The objectives of this study are to confirm whether these five mycotoxins were present in the cranes’ food and to discuss the potential for mycotoxin impact on the health of red-crowned cranes in the Yancheng Biosphere Reserve, China.

Peripheral blood of red-crowned crane (♀) were collected from Beijing Zoo in Beijing, China. The specimens were kept in the laboratory at −80 °C, total genomic DNA was extracted using a DNA extract kit following the previously reports (Bai et al. 2010; Gunal et al. 2018). And we used next-generation sequencing to perform low coverage whole genome sequencing. Initially, the raw next-generation sequencing reads generated from Illumina HiSeq PE150 (Illumina, San Diego, CA). 41,442,450 clean reads were de novo assembly by using commercial software (Geneious V9, Auckland, New Zealand) to produce a single, circular form of complete mitogenome. The total length of its mitogenome was 16,727 bp, with a genome size similar to other research (Krajewsk et al. 2010), but more details on that study. Compared with the current mitogenome sequences of red-crowned crane, there were 12 bases insertion, and 374 bases substitution in the red-crowned crane mitogenome of China we sequenced. The accurate annotated mitochondrial genome sequence was submitted to GeneBank with accession number MH041485. The complete mitogenome of red-crowned crane was 16,727 bp in size and its overall base composition was 31.2% for A, 31.3% for C, 13.6% for G, and 23.9% for T, and had GC content of 43.82%.

The protein coding, rRNA and tRNA genes of red-crowned crane mitogenome were predicted by using DOGMA (Wyman et al. 2004), ARWEN (Laslett and Canback 2008), MITOS (Bernt et al. 2013) tools and manually inspected. The complete mitogenome of red-crowned crane includes unique 14 protein-coding genes (PCGs), 22 transfer RNA genes, and two ribosomal RNA genes, all details were listed in GeneBank (MH041485).

To further validate the new sequences, we used all of mitochondrial genome sequences published in GeneBank of other crane species to construct the phylogenetic tree. These species were as follows: Struthio camelus, Grus japonensis, Grus Americana, Grus grus, Grus monacha, Grus nigricollis, Grus carunculatus, Anthropoides paradiseus, Grus vipio, Grus rubicunda, Grus Antigone, Grus Canadensis, and Grus leucogeranus. We used MEGA6 to produce the phylogenetic tree based on the maximum-likelihood method (Figure 1). The phylogenetic analysis results support that the mitochondrial DNAs of red-crowned crane are more closely related to other species of crane genus.

**Figure 1.**
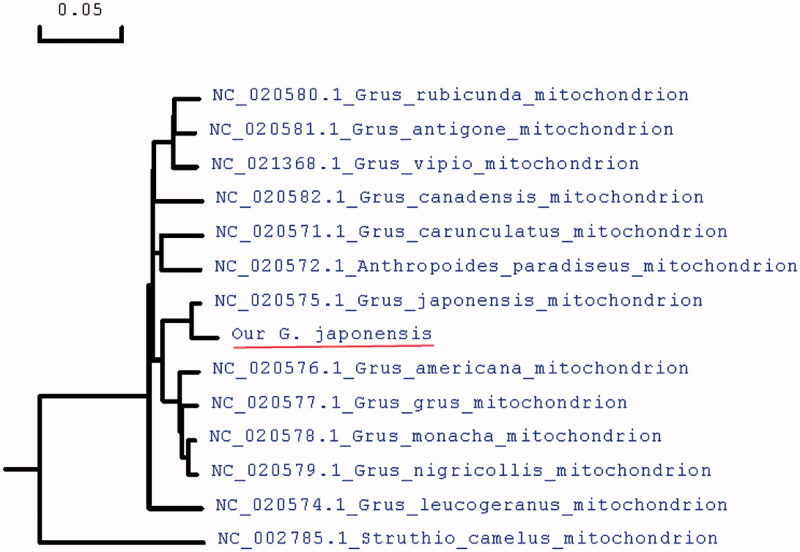
Molecular phylogeny of red-crowned crane and related species in Crane based on complete mitogenome. The complete mitogenomes are downloaded from GenEBank and the phylogenetic tree is constructed by maximum-likelihood method with 500 bootstrap replicates. The gene’s accession number for tree construction is listed in front of the species name.
